# An AGEF-1/Arf GTPase/AP-1 Ensemble Antagonizes LET-23 EGFR Basolateral Localization and Signaling during *C. elegans* Vulva Induction

**DOI:** 10.1371/journal.pgen.1004728

**Published:** 2014-10-16

**Authors:** Olga Skorobogata, Juan M. Escobar-Restrepo, Christian E. Rocheleau

**Affiliations:** 1 Division of Endocrinology and Metabolism, Department of Medicine, Research Institute of the McGill University Health Centre, McGill University, Montreal, Quebec, Canada; 2 Department of Anatomy and Cell Biology, McGill University, Montreal, Quebec, Canada; 3 Institute of Molecular Life Sciences, University of Zürich, Zürich, Switzerland; University of California San Diego, United States of America

## Abstract

LET-23 Epidermal Growth Factor Receptor (EGFR) signaling specifies the vulval cell fates during *C. elegans* larval development. LET-23 EGFR localization on the basolateral membrane of the vulval precursor cells (VPCs) is required to engage the LIN-3 EGF-like inductive signal. The LIN-2 Cask/LIN-7 Veli/LIN-10 Mint (LIN-2/7/10) complex binds LET-23 EGFR, is required for its basolateral membrane localization, and therefore, vulva induction. Besides the LIN-2/7/10 complex, the trafficking pathways that regulate LET-23 EGFR localization have not been defined. Here we identify *vh4*, a hypomorphic allele of *agef-1*, as a strong suppressor of the *lin-2* mutant Vulvaless (Vul) phenotype. AGEF-1 is homologous to the mammalian BIG1 and BIG2 Arf GTPase guanine nucleotide exchange factors (GEFs), which regulate secretory traffic between the Trans-Golgi network, endosomes and the plasma membrane via activation of Arf GTPases and recruitment of the AP-1 clathrin adaptor complex. Consistent with a role in trafficking we show that AGEF-1 is required for protein secretion and that AGEF-1 and the AP-1 complex regulate endosome size in coelomocytes. The AP-1 complex has previously been implicated in negative regulation of LET-23 EGFR, however the mechanism was not known. Our genetic data indicate that AGEF-1 is a strong negative regulator of LET-23 EGFR signaling that functions in the VPCs at the level of the receptor. In line with AGEF-1 being an Arf GEF, we identify the ARF-1.2 and ARF-3 GTPases as also negatively regulating signaling. We find that the *agef-1(vh4)* mutation results in increased LET-23 EGFR on the basolateral membrane in both wild-type and *lin-2* mutant animals. Furthermore, *unc-101(RNAi)*, a component of the AP-1 complex, increased LET-23 EGFR on the basolateral membrane in *lin-2* and *agef-1(vh4); lin*-2 mutant animals. Thus, an AGEF-1/Arf GTPase/AP-1 ensemble functions opposite the LIN-2/7/10 complex to antagonize LET-23 EGFR basolateral membrane localization and signaling.

## Author Summary

In the nematode, *Caenorhabditis elegans*, an evolutionarily conserved Epidermal Growth Factor Receptor (EGFR) signaling pathway is required to induce three epithelial cells to initiate a program of vulva development. EGFR on the basolateral membrane is essential to engage and transmit this signal. Here we demonstrate that AGEF-1 and the AP-1 clathrin adaptor complex function with two Arf GTPases to regulate EGFR localization and signaling. In humans, EGFR also localizes to the basolateral membrane of epithelial cells, and excessive EGFR signaling is a major driver of cancer. In *C. elegans*, we show that loss of AGEF-1 results in an increase in basolateral EGFR localization in the vulva precursor cells, and in sensitized genetic backgrounds, a corresponding increase in vulva induction. While the human AGEF-1 proteins, BIG1 and BIG2, have not been previously implicated in EGFR signaling and cancer, mutations in BIG2 are causal of periventricular heterotopia, a condition whereby neurons fail to migrate to the cerebral cortex during brain development. As migrating neurons require polarized protein localization, BIG2 and AGEF-1 may have similar functions in these polarized cell types.

## Introduction

*C. elegans* vulval cell induction requires a highly conserved Epidermal Growth Factor Receptor (EGFR)/Ras GTPase/Mitogen Activated Protein Kinase (MAPK) signaling pathway providing an *in vivo* model in which to study signaling in a polarized epithelia [1], [2]. During larval development, an equivalence group of six vulval precursor cells (VPCs), P3.p-P8.p, have the potential to be induced to generate the vulva. The anchor cell in the overlying gonad secretes the LIN-3 EGF-like ligand, inducing the closest VPC, P6.p, to adopt the primary vulval fate, and a combination of graded LIN-3 EGF signal and lateral signaling by LIN-12 Notch specifies the neighboring VPCs, P5.p and P7.p, to adopt the secondary vulval fate. Together P5.p-P7.p generate the 22 nuclei of the mature vulva, eight cells from the primary cell and seven from each of the secondary cells. The remaining VPCs, P3.p, P4.p, and P8.p, divide once and fuse with the surrounding hypodermal syncytium (50% of the time P3.p fuses prior to dividing) and thus adopt a tertiary non-vulval fate. Inhibition of LET-23 EGFR signaling causes a Vulvaless (Vul) phenotype in which less than the normal three VPCs are induced. Conversely, increased LET-23 EGFR signaling causes a Multivulva (Muv) phenotype in which greater than three VPCs are induced.

LET-23 EGFR localizes to both the apical and basolateral membranes of the VPCs, though, it is the basolateral localization that is thought to engage LIN-3 EGF and induce vulva induction [3], [4], [5]. A tripartite complex of proteins, LIN-2 Cask, LIN-7 Veli, and LIN-10 Mint (LIN-2/7/10), interacts with the C-terminal tail of LET-23 EGFR and is required for its basolateral localization [3], [4]. Mutations in any component of the complex, or the *let-23(sy1)* mutation, which deletes the last six amino acids of LET-23 EGFR that are required for its interaction with LIN-7, result in LET-23 EGFR localizing only to the apical membrane and a strong Vul phenotype [3], [4], [6], [7], [8]. The Vul phenotype of *lin-2/7/10* mutants or the *let-23(sy1)* mutant are easily suppressed to a wild-type or even a Muv phenotype by loss of negative regulators of LET-23 EGFR signaling such as *sli-1 Cbl, gap-1 RasGAP, rab-7 GTPase*, and *unc-101 AP-1μ* [5], [9], [10], [11], [12]. Thus far, no suppressors of the *lin-2/7/10* mutant Vul phenotype have been shown to restore LET-23 EGFR to the basolateral membrane.

UNC-101 and APM-1 are two μ1 subunits for the AP-1 adaptor protein complex, which function redundantly to antagonize vulva cell induction [12], [13]. In mammals, AP-1 localizes to the *trans*-Golgi network (TGN) and endosomes, promotes formation of clathrin-coated vesicles, and is involved in regulated secretion from the TGN. [14], [15], [16]. In epithelial cells, AP-1 sorts cargo, including EGFR, to the basolateral membrane, which would be inconsistent with AP-1 antagonizing signaling [17], [18]. The small GTPase, Arf1, recruits AP-1 to the TGN and thus facilitates the formation of clathrin-coated vesicles [15], [19], [20]. BIG1 and BIG2 are Sec7 domain containing guanine nucleotide exchange factors (GEFs) for class I Arf GTPases [21], [22], and are required for recruitment of AP-1 to the TGN and endosomes [23], [24]. To date, neither Arf1 nor the BIG1/2 GEFs have been implicated in EGFR/Ras/MAPK signaling.

Here we identify *C. elegans* AGEF-1, a homolog of yeast Sec7p and the mammalian BIG1 and BIG2 Arf GEFs, as negatively regulating EGFR/Ras/MAPK-mediated vulva induction. We show that AGEF-1 regulates protein secretion in multiple tissues, regulates polarized localization of the SID-2 transmembrane protein in the intestine, and regulates the size of late endosomes/lysosomes with the AP-1 complex in the macrophage/scavenger cell-like coelomocytes. Genetic epistasis places AGEF-1 upstream or in parallel to LET-23 EGFR. We find that the ARF-1.2 and ARF-3 GTPases also negatively regulate LET-23 EGFR signaling. Moreover, our genetics are consistent with AGEF-1 BIG1/2, ARF-1.2 Arf1 and UNC-101 AP-1μ1 functioning together in preventing ectopic vulva induction. It has been 20 years since UNC-101 was identified as a negative regulator of LET-23 EGFR signaling, however its mechanism of action has remained an enigma [12]. Contrary to the role of AP-1 in basolateral sorting in mammalian cells, we demonstrate that AGEF-1 BIG1/2 and UNC-101 AP-1μ1 antagonize the basolateral membrane localization of LET-23 EGFR in the VPCs. Thus, the AGEF-1/Arf GTPase/AP-1 ensemble antagonizes LET-23 EGFR-mediated vulva induction via regulation of LET-23 EGFR membrane localization.

## Results

### Identification of *agef-1(vh4)* as a suppressor of the *lin-2(e1309)* Vul phenotype

**Figure 1:**
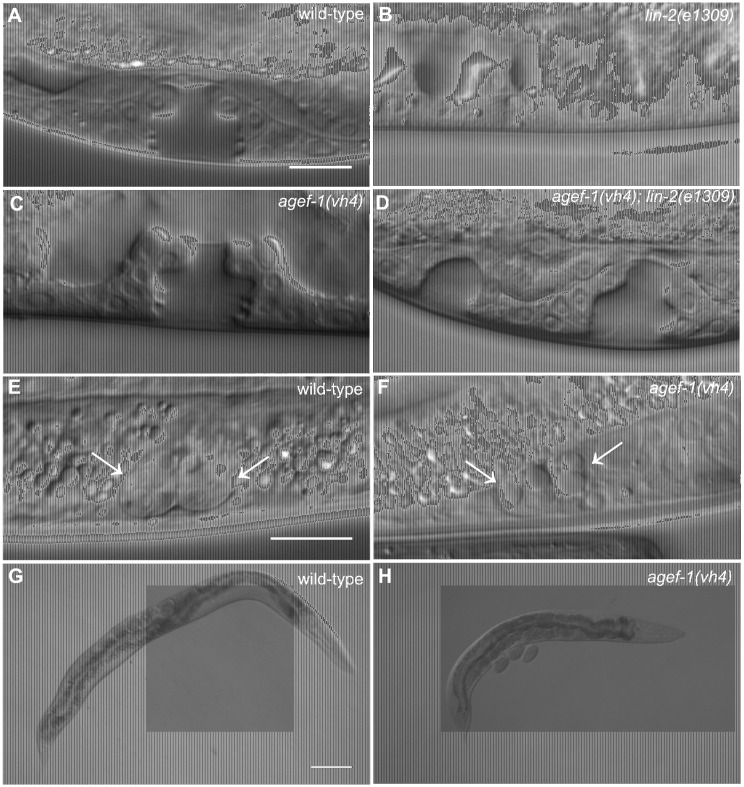
*agef-1(vh4*) suppresses the *lin-2(e1309)* Vul phenotype, has large vesicles in coelomocytes and a dumpy body morphology. (A–D) Representative Differential Interference Contrast (DIC) images of vulvas of wild-type, *lin-2(e1309)*, *agef-1(vh4)* and *agef-1(vh4); lin-2(e1309)* L4 stage larvae. The *lin-2(e1309)* larva lacks a vulva while *agef-1(vh4); lin-2(e1309)* has a second vulval invagination and hence is Muv. *agef-1(vh4)* single mutants have a wild-type vulva. Bar, 10 µm. (E, F) DIC images of coelomocyte pairs (white arrows) of wild-type and *agef-1(vh4)* L4 larvae. *agef-1(vh4)* coelomocytes have enlarge vesicles as compared to wild-type. Bar, 10 µm. (G, H) DIC images of adult wild-type and *agef-1(vh4)* animals; *agef-1(vh4)* mutants have a smaller body length compared to wild-type. Bar, 100 µm.

We previously reported that *rab-7(ok511)*, a maternal effect embryonic lethal mutant, strongly suppresses the *lin-2(e1309)* Vul phenotype [11]. To identify new candidate regulators of LET-23 EGFR trafficking and signaling we conducted a clonal screen for essential suppressors of *lin-2(e1309)* (see Materials and Methods). In this screen we identified *vh4* as a strong suppressor of the *lin-2(e1309)* Vul phenotype (Figure 1A–D; Table 1, lines 1–4). The *vh4* mutation can suppress the 100% Vul phenotype of *lin-2(e1309)* to 20% Vul, and 30% Muv. In a *lin-2(+)* background, however, *vh4* mutant animals have 100% wild-type vulva induction. Consistent with a potential role in vesicular trafficking, the coelomocytes (macrophage-like scavenger cells) of *vh4* mutants accumulate abnormally large vesicular structures (Figure 1E, F). Additionally, *vh4* mutants have a dumpy body morphology, uncoordinated movement and ∼50 percent embryonic lethality (Figure 1G, H).

**Table 1:** AGEF-1 is a negative regulator of EGFR/Ras/MAPK signaling during vulva induction.

	GENOTYPE	Muv, %	Vul, %	AVG. # of VPCs INDUCED	VPCs INDUCED, %						*n*
					P3.p	P4.p	P5.p	P6.p	P7.p	P8.p	
1	wild-type	0	0	3.0	0	0	100	100	100	0	many
2	*agef-1(vh4)*	0	0	3.0	0	0	100	100	100	0	40
3	*lin-2(e1309)*	0	100	0.31	0	1	6	13	11	0	35
4	*agef-1(vh4); lin-2(e1309)*	30***	20****	3.0****	0	15	90****	98****	88****	6	40
5	*agef-1(vh4); lin-2(e1309); vhEx7*	5*	90****	1.69****	0	2	43***	77**	41***	5	21
6	*agef-1(vh4); lin-2(e1309); vhEx8*	4*	61**	2.07****	0	0	61**	93	50**	2	23
7	*agef-1(RNAi); lin-2(e1309)*	0	90	0.93***	0	3	25*	38*	28	0	40
8	*zhIs035; lin-2(e1309)*	0	0****	3.0****	0	0	100****	100****	100****	0	23
9	*zhIs038; lin-2(e1309)*	8	12****	2.96****	0	4	100****	96****	96****	0	25
10	*let-60(n1046)*	65	0	3.55	33	15	100	100	100	8	20
11	*agef-1(vh4); let-60(n1046)*	88**	0	4.04	19	51**	100	100	100	34	40
12	*let-23(sy1)*	0	100	0.14	0	0	9	0	5	0	40
13	*agef-1(vh4); let-23(sy1)*	4	48****	2.3****	0	4	66****	90****	64****	5	50
14	*let-23(sy97)*	0	93	0.4	0	0	14	16	10	0	29
15	*agef-1(vh4); let-23(sy97)*	0	96	0.24	0	0	7	11	6	0	27
16	*lin-3(e1417)*	0	98	0.72	0	0	13	48	11	0	80
17	*agef-1(vh4); lin-3(e1417)*	0	76**	1.54****	0	0	38**	78***	36**	2	50
18	*sli-1(sy143)*	0	0	3.0	0	0	100	100	100	0	31
19	*agef-1(vh4); sli-1(sy143)*	59****	0	3.42***	0	0	98	100	100	44****	32
20	*unc-101(sy108)*	0	0	3.0	0	0	100	100	100	0	25
21	*unc-101(RNAi) agef-1(vh4)*	43****	0	3.38***	0	1	100	100	100	37****	42
22	*unc-101(sy108) agef-1(RNAi)*	54****	0	3.34**	0	12*	100	100	100	22**	37
23	*arf-1.2(ok796)*	0	0	3.0	0	0	100	100	100	0	25
24	*unc-101(sy108); arf-1.2(ok796)*	50****	0	3.35***	0	0	100	100	100	35***	50
25	*agef-1(vh4); arf-1.2(ok796)*	63****	5	3.29**	0	0	94	94	94	34****	19

Statistical analysis was performed using Fisher's exact test (www.graphpad.com/quickcalcs) comparing each double mutant with the single mutant on the line above with the following exceptions: lines 5 and 6 were compared to line 4, lines 7, 8 and 9 were compared to line 3, and lines 19, 21, 22, 24, and 25 were compared to line 2.

*P<0.05;

**P<0.01;

***P<0.001;

****P<0.0001.

**Figure 2:**
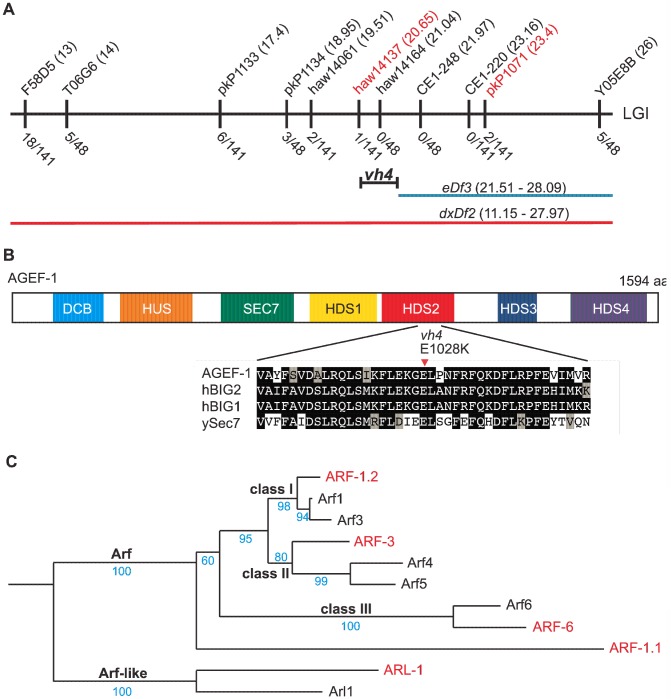
*vh4* is a missense mutation in *agef-1* and homology between *C. elegans* and human Arf GTPases. (A) Schematic representation of the right end of chromosome I (LGI). The SNPs used for interval mapping are indicated on top with their chromosomal locations (map units). The number of recombinant animals positive for the Hawaiian SNP out of the total number of animals tested for each SNP is indicated below. The two chromosomal deficiencies used for mapping are *dxDf2* and *eDf3* shown in red and blue, respectively. A bracket indicates the 0.9 map unit interval between the *haw14137* SNP and the left end of *eDf3* to which *vh4* maps. (B) Homology domains of the AGEF-1 protein that are common with the human BIG1 and BIG2 proteins and *S. cerevisiae* Sec7 Arf GEFs: Dimerization Cyclophilin Binding (DCB), Homology Upstream of Sec7 (HUS), catalytic GEF domain (SEC7), Homology Downstream of Sec7 (HDS1-4). HDS1-4 are not homologous to each other. Below shows the alignment of the sequences around the amino acid E1028 which is substituted for a Lysine (K) in *agef-1(vh4)*. E1028 is conserved in the human and yeast homologs. Amino acid (aa) identities are highlighted in black and similarities in grey. (C) A phylogenetic tree showing the evolutionary relationship between the four *C. elegans* Arf GTPases and the three classes of human Arf GTPases as well as the closely related Arl1 GTPases. The human and *C. elegans* Arfs are depicted in black and red, respectively. Bootstraps are shown in blue.

To determine the molecular identity of *vh4*, we used a single nucleotide polymorphism (SNP) mapping strategy [25]. Genome-wide mapping located *vh4* to the right arm of chromosome I, and interval mapping placed *vh4* in a 2.75 map unit region between SNPs *haw14137* and *pkP1071* at positions 20.65 and 23.4 map units, respectively (Figure 2A). We further refined the genomic interval by complementation with chromosomal deficiencies *dxDf2* and *eDf3*. *vh4* failed to complement the large deficiency *dxDf2*, but complemented the small *eDf3*, indicating that *vh4* lies in a 0.9 map unit region (20.65–21.51) containing 27 genes. We found that one obvious candidate, *vps-28*, was an RNAi suppressor of the *lin-2* Vul phenotype [11]. However, *vh4* complemented the *vps-28(tm3767)* deletion allele and no lesion in the *vps-28* coding sequence of *vh4* animals was detected by DNA sequencing suggesting that *vh4* is not an allele of *vps-28*. Whole genome sequencing revealed a homozygous G to A transition at position 3082 in exon 11 of the *agef-1* gene (AAA TTT TTG GAA AAG GGA **G**AA CTT CCG AAT TTC CGA TTT) that corresponds to a glutamate to lysine substitution in a conserved region of the predicted AGEF-1 protein (Figure 2B). Consistent with *vh4* being a mutation in *agef-1*, we find that *agef-1(RNAi)* suppresses the severity of the *lin-2* Vul phenotype (Table 1, lines 3 and 7) and *agef-1(vh4)* mutant oocytes have defects in CAV-1 body formation as previously seen with *agef-1(RNAi)* (Figure S1J–M) [26]. Finally, *agef-1(vh4)* fails to complement two deletion alleles, *agef-1(ok1736)* and *agef-1(tm1693)*, resulting in a strong embryonic lethal phenotype. These data indicate that *vh4* is a hypomorphic allele of *agef-1*.

### The coelomocytes of *agef-1(vh4)* mutants have enlarged late endosomes/lysosomes

**Figure 3:**
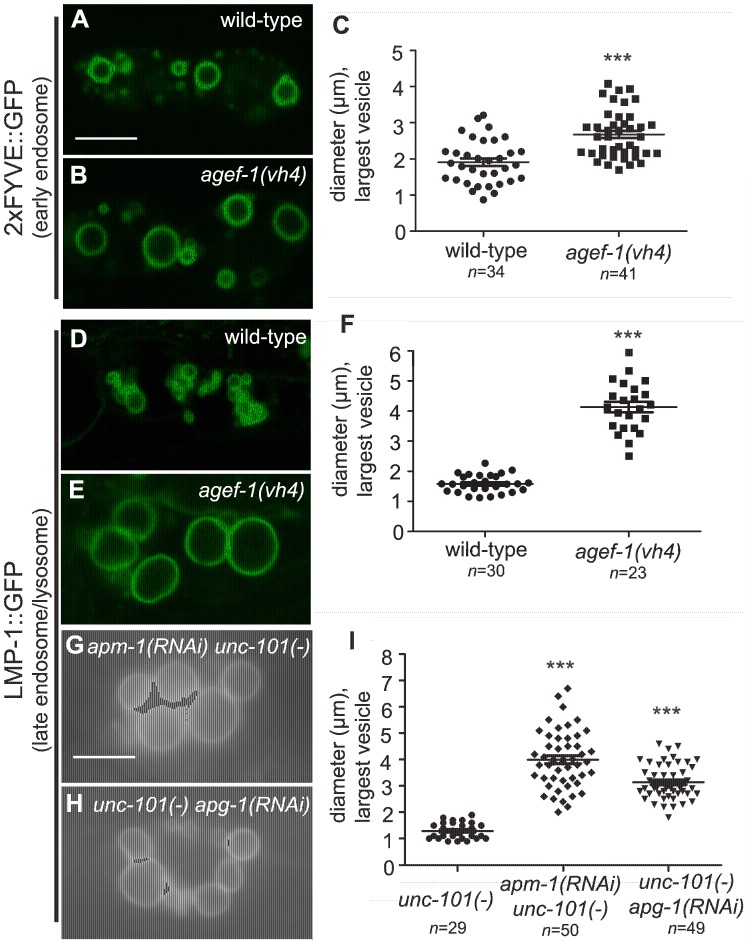
AGEF-1 and the AP-1 complex regulate the size of late endosomes/lysosomes in coelomocytes. (A, B) Confocal images of the coelomocytes of wild-type and *agef-1(vh4)* L4 larvae expressing the early endosomal marker 2×FYVE::GFP. (C) Quantification of the diameter of the largest 2×FYVE::GFP-positive vesicle per coelomocyte in wild-type and *agef-1(vh4)*. (D, E) Confocal images of the coelomocytes of wild-type and *agef-1(vh4)* L4 larvae expressing the late endosomal/lysosomal marker LMP-1::GFP. (F) Quantification of the diameter of the largest LMP-1::GFP-positive vesicle per coelomocyte in wild-type and *agef-1(vh4)*. (G, H) Epifluorescent images of the coelomocytes of *apm-1(RNAi) unc-101(sy108)* and *unc-101(sy108) apg-1(RNAi)* L4 larvae. (I) Quantification of the diameter of the largest LMP-1::GFP-positive vesicle per coelomocyte upon depletion of multiple AP-1 subunits. Prism 5 (GraphPad Software, Inc., La Jolla, CA) was used for statistical analysis; unpaired t-test was performed to compare changes in the vesicle size. *** *P<0.001*. Shown is the mean vesicle size plus standard error of the mean. All bars, 5 µm.

To determine the identity of the large vesicles in *agef-1(vh4)* coelomocytes, we used GFP tagged endosomal and Golgi proteins. Since the vesicles are presumably in flux, we measured the diameter of the largest GFP-positive vesicle per coelomocyte. We found a modest, but significant increase in the size of vesicles positive for the early endosomal 2×FYVE::GFP and the pan-endosomal RME-8::GFP markers in *agef-1(vh4)* animals as compared to wild-type (Figure 3A–C and Figure S2A–C). However, the large vesicles in *agef-1(vh4)* coelomocytes visible by DIC optics correspond to LMP-1::GFP, a marker for late endosomes/lysosomes (Figure 3D–F). This finding corroborates a concurrent study identifying large LMP-1 positive vesicles in the coelomocytes of *agef-1(RNAi)* animals [27]. LMP-1 is a transmembrane protein, whose mammalian homolog, Lamp1, can transit from Golgi via the plasma membrane and endosomes to the lysosome [28]. Therefore, we assessed the morphology of the Golgi in *agef-1(vh4)* mutants using a mannosidase II::GFP marker. While there might be a slight increase in the size of the Golgi mini-stacks, they were distinct from the large LMP-1::GFP positive vesicles seen by DIC (Figure S2D–F). Of note, the mannosidase II::GFP strands that appear to interconnect the Golgi mini-stacks in wild-type (30/37 coelomocytes) were largely absent in *agef-1(vh4)* mutants (6/42 coelomocytes are interconnected). These data show that *agef-1(vh4)* disrupts endosome and Golgi morphology and possibly trafficking.

### The body wall muscle cells and intestinal cells of *agef-1(vh4)* mutants have defects in protein secretion

**Figure 4:**
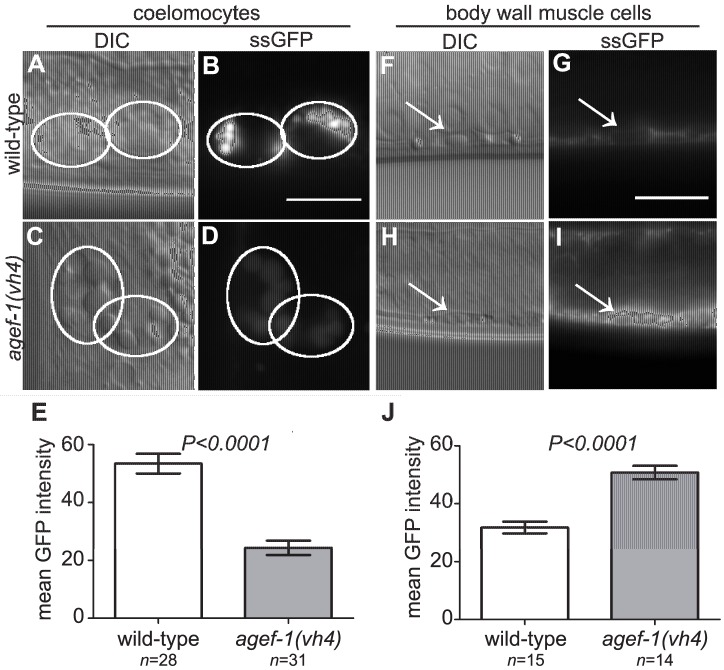
Secretion defect from the body wall muscle cells in *agef-1(vh4)* mutants. (A–D) Representative DIC and epifluorescent images of ssGFP in the coelomocytes of wild-type and *agef-1(vh4)* mutants. Coelomocytes are outlined with white circles. (E) Quantification of mean ssGFP pixel intensity in the coelomocytes. Images were acquired at an exposure time of 25 ms. (F–I) Representative DIC and epifluorescent images of wild-type and *agef-1(vh4)* body wall muscle cells expressing ssGFP. Arrows indicate nuclei of the muscle cells. (J) Quantification of mean ssGFP pixel intensity in the body wall muscle cells. Images were acquired at an exposure time of 100 ms. Prism 5 (GraphPad Software, Inc., La Jolla, CA) was used for statistical analysis; unpaired t-test was performed to compare mean GFP intensities and standard error of the mean between wild-type and *agef-1(vh4)* animals. All bars, 10 µm.

To test if *agef-1(vh4)* coelomocytes have an endocytosis defect we analyzed the internalization of a signal secreted GFP (ssGFP) that is expressed in body wall muscle cells, secreted into the pseudocoelom, and endocytosed by the coelomocytes [29]. We found less ssGFP in the coelomocytes of *agef-1(vh4)* animals as compared to wild-type (Figure 4A–E). However, we did not detect a significant accumulation of ssGFP in the pseudocoelom of *agef-1(vh4)* mutants as would be expected for an endocytosis defect. Rather there was a clear accumulation of ssGFP in the body wall muscle cells of *agef-1(vh4)* animals as compared to wild-type (Figure 4F–J). While this does not rule out a potential endocytosis defect in *agef-1(vh4)* coelomocytes, it does indicate that *agef-1(vh4)* mutants have a secretion defect in the body wall muscle cells.

We also analyzed a Yolk::GFP fusion (YP170::GFP) that is secreted from the intestine, and internalized by maturing oocytes [30]. We did not detect a difference in the uptake of YP170::GFP by oocytes (Figure S1A–D), however YP170::GFP levels in the intestine were higher in *agef-1(vh4)* animals than in wild-type (Figure S1E–I). An independent study also found impaired secretion of yolk in *agef-1(RNAi)* animals [31]. Thus, *agef-1(vh4)* mutants have impaired protein secretion from both body wall muscle and intestinal cells.

### AGEF-1 antagonizes signaling upstream or in parallel to LET-23 EGFR

To understand the role of AGEF-1 in the LET-23 EGFR/LET-60 Ras signaling pathway, we made double mutants with *agef-1* and several mutations in core components of the pathway. A gain of function mutation in *let-60 ras (n1046)* causes a Muv phenotype that can be enhanced by loss of a negative regulator of the pathway [11], [32], [33], [34]. *agef-1(vh4)* significantly enhances the Muv phenotype of *let-60(n1046)*, consistent with AGEF-1 being a negative regulator of signaling (Table 1, lines 10–11). We performed epistasis analysis to determine at which step of the pathway AGEF-1 functions. We found that *agef-1(vh4)* strongly suppresses the Vul phenotype of the *let-23(sy1)* mutant (Table 1, lines 12–13). The *sy1* allele truncates the last six amino acids of LET-23 EGFR that are required for its interaction with the LIN-2/7/10 complex, and thus behaves identical to mutations in components of this complex [3], [8]. However, *agef-1(vh4)* fails to suppress the Vul phenotype of the *let-23(sy97)* allele that results in a more severe truncation of LET-23 EGFR that blocks signaling to the LET-60 Ras (Table 1, lines 14–15) [7]. We next tested if *agef-1(vh4)* can suppress the Vul phenotype of *lin-3(e1417)*, a strong hypomorphic allele of *lin-3 EGF* [35]. We found that *agef-1(vh4)* partially suppressed the *lin-3(e1417)* Vul phenotype (Table 1, lines 16–17). These data are consistent with AGEF-1 antagonizing signaling upstream or in parallel to LET-23 EGFR.

SLI-1 Cbl, a putative E3-ubiquitin ligase, and UNC-101 AP-1μ are negative regulators of LET-23 EGFR signaling that also function at the level of LET-23 EGFR [9], [12], [36]. Like *agef-1(vh4)*, mutations in *sli-1 Cbl* and *unc-101 AP-1μ* do not cause a vulval phenotype alone, but double mutants cause a synergistic Muv phenotype. Therefore, we tested if *agef-1(vh4)* is Muv in combination with strong loss-of-function alleles of *sli-1 Cbl* and *unc-101 AP-1μ.* We found that *agef-1(vh4); sli-1(sy143)* animals are strongly Muv, suggesting that AGEF-1 might function in parallel to SLI-1 Cbl (Table 1, lines 18–19). We were unable to identify *unc-101(sy108) agef-1(vh4)* double mutants segregating from *unc-101(sy108) agef-1(vh4)/unc-101(sy108)* mothers suggesting that they are zygotic lethal. Thus, we fed L1 larvae RNAi and found that both *unc-101(RNAi) agef-1(vh4)* and *unc-101(sy108) agef-1(RNAi)* animals have a strong Muv phenotype (Table 1, lines 20–22). Since there are two AP-1µ genes, *unc-101* and *apm-1*, that are functionally redundant, we cannot conclude whether AGEF-1 functions in parallel to UNC-101, or whether they function together; we favor the later, see below. However, the strong genetic interactions of *agef-1(vh4)* and mutations in *sli-1* and *unc-101* further support AGEF-1 functioning at the level of LET-23 EGFR to negatively regulate signaling.

### ARF-1.2 and ARF-3 antagonize LET-23 EGFR signaling

The identification of a putative Arf GEF as a negative regulator of LET-23 EGFR signaling suggests that one or more of the four *C. elegans* Arf GTPases might also regulate LET-23 EGFR signaling. The mammalian Arf GTPases have been placed in three classes based on homology [37]. To gain a better understanding of the relationship of the *C. elegans* and human Arf GTPases we undertook a phylogenetic analysis (Figure 2C). From this we conclude that *C. elegans* ARF-1.2 is homologous to Class I Arfs; ARF-3 is related to both Class I and II Arfs, but clusters with the Class II; ARF-6 is a homolog of the Class III Arf, whereas ARF-1.1 appears to be a Caenorhabiditis specific Arf GTPase that is distinct from the Arf-like Arl GTPases (Figure 2C). We used RNAi and deletion mutants to test each *arf* gene for suppression of the *lin-2(e1309)* Vul phenotype. RNAi of either *arf-1.2* or *arf-3* partially suppressed of the *lin-2(e1309)* Vul phenotype (Table 2, lines 1–3). The *arf-1.2(ok796)* deletion mutant was a much more potent suppressor of the *lin-2(e1309)* Vul phenotype consistent with RNAi being less effective in the VPCs [11; this study] (Table 2, lines 4–5). The *arf-3(tm1877)* deletion is zygotic lethal and did not permit analysis. However, *arf-3(RNAi)* into *arf-1.2(ok796); lin-2(e1309)* animals led to an even stronger suppression of the Vul phenotype comparable to that of *agef-1(vh4); lin-2(e1309)* (Table 2, line 8). Neither the *arf-6(tm1447)* nor the *arf-1.1(ok1840)* deletions were able to suppress the *lin-2(e1309)* Vul phenotype (Table 2, lines 9–12). These data suggest that ARF-1.2 and ARF-3 function in a partly redundant manner, possibly with AGEF-1, to antagonize LET-23 EGFR signaling.

**Table 2:** Class I and II Arf mutants suppress the *lin-2(e1309)* Vul phenotype.

	GENOTYPE	Muv%	Vul%	AVG. # of VPCs INDUCED	VPCs INDUCED, %						*n*
					P3.p	P4.p	P5.p	P6.p	P7.p	P8.p	
1	*lin-2(e1309)*	0	100	0.31	0	1	6	13	11	0	35
2	*arf-1.2(RNAi); lin-2(e1309)*	0	95	0.73**	0	0	18	30	25	0	40
3	*arf-3(RNAi); lin-2(e1309)*	0	85*	0.79**	0	1	25*	23	30	0	40
4	*arf-1.2(ok796)*	0	0	3.0	0	0	100	100	100	0	25
5	*arf-1.2(ok796); lin-2(e1309)*	20***	52****	2.66****	0	30**	85****	100****	51***	0	40
6	*arf-1.2(ok796); lin-2(e1309); vhEx7*	0*	90**	1.48****	0	0**	38***	70***	40	0	20
7	*arf-1.2(ok796); lin-2(e1309); vhEx8*	0	100**	1.38****	0	0*	33***	67**	38	0	12
8	*arf-1.2(ok796); arf-3(RNAi); lin-2(e1309)*	34****	25****	2.97****	0	23*	89****	98****	72****	16*	44
9	*arf-6 (tm1447)*	0	0	3.0	0	5	100	100	95	0	22
10	*arf-6(tm1447); lin-2(e1309)*	0	94	0.4	0	0	16	13	11	0	35
11	*arf-1.1(ok1840)*	0	0	3.0	0	0	100	100	100	0	20
12	*arf-1.1(ok1840); lin-2(e1309)*	0	97	0.37	0	1	11	17	7	0	35

Statistical analysis was performed as described above. All mutant combinations with *lin-2(e1309)* were compared to *lin-2(e1309)* single mutants except for *arf-1.2(ok796); lin-2(e1309); vhEx7* and *arf-1.2(ok796); lin-2(e1309); vhEx8*, which were compared to *arf-1.2(ok796); lin-2(e1309)*.

*P<0.05;

**P<0.01;

***P<0.001;

****P<0.0001.

### ARF-1.2 and AGEF-1 antagonize LET-23 EGFR signaling in the VPCs

To test if *arf-1.2* was required in the VPCs we generated two transgenic extrachromosomal arrays, *vhEx7* and *vhEx8*, expressing ARF-1.2::GFP under the control of the VPC-specific promoter, *lin-31* [38]. Both transgenic lines were able to strongly rescue *arf-1.2(ok796)* suppression of the *lin-2(e1309)* Vul phenotype (Table 2, lines 6–7). Thus, ARF-1.2 functions in the VPCs to negatively regulate LET-23 EGFR signaling. We next tested whether VPC-specific overexpression of ARF-1.2::GFP can revert the suppression of Vul phenotype in *agef-1(vh4); lin-2(e1309)*. Both lines, *vhEx7* and *vhEx8*, led to a more severe Vul phenotype when expressed in *agef-1(vh4); lin-2(e1309)* animals (Table 1, lines 5–6). This suggests that AGEF-1 functions in the VPCs through ARF-1.2 to antagonize LET-23 EGFR signaling.

### AGEF-1, ARF-1.2, and UNC-101 AP-1*μ* cooperate to repress ectopic vulva induction

Having observed a strong Muv phenotype in *unc-101(RNAi) agef-1(vh4)* and *unc-101(sy108) agef-1(RNAi)* doubles (Table 1, lines 21–22), we hypothesized that *arf-1.2(ok796)* would have similar interactions. Indeed, both *unc-101(sy108); arf-1.2(ok796)* and *agef-1(vh4); arf-1.2(ok796)* animals have a strong Muv phenotype (Table 1, lines 24–25). Given that *agef-1(vh4)* is a weak hypomorphic allele and ARF-1.2 and UNC-101 AP-1μ are each functionally redundant with ARF-3 and APM-1 AP-1μ, respectively; these data are consistent with AGEF-1, ARF-1.2 and UNC-101 AP-1μ functioning together to negatively regulate LET-23 EGFR signaling.

### AGEF-1 and UNC-101 have shared phenotypes in coelomocytes and the intestine

If AGEF-1, the ARF GTPases and the AP-1 complex function together, we expect that they will have shared phenotypes. We tested whether the ARFs and the AP-1 complex regulate the size of vesicles in coelomocytes as does AGEF-1. While *unc-101(sy108)* mutants do not have large vesicles, further depletion of the AP-1 complex by RNAi of *apm-1 AP-1μ* or *apg-1 AP-1γ* in the *unc-101(sy108)* background resulted in enlarged LMP-1::GFP vesicles in the coelomocytes (Figure 3G–I). Consistent with previous studies, we found no evidence for *arf-1.2* or *arf-3* in regulating the size of vesicles in the coelomocytes [27], nor do deletions in *arf-1.1* or *arf-6*. The complement of ARF GTPases that function with AGEF-1 and AP-1 in coelomocytes remains to be determined.

The AP-1 complex has recently been shown to restrict both apical and basolateral membrane protein localization in the *C. elegans* intestine [39], [40]. Similarly, we found that the apically localized SID-2 transmembrane protein [41], was mislocalized to the cytoplasm and basolateral membranes in *agef-1(vh4)* mutants (Figure S3A–D), suggesting that AGEF-1 and AP-1 might function together to regulate polarized localization of membrane proteins in the intestine.

### AGEF-1 and UNC-101 antagonize LET-23 EGFR basolateral localization

**Figure 5:**
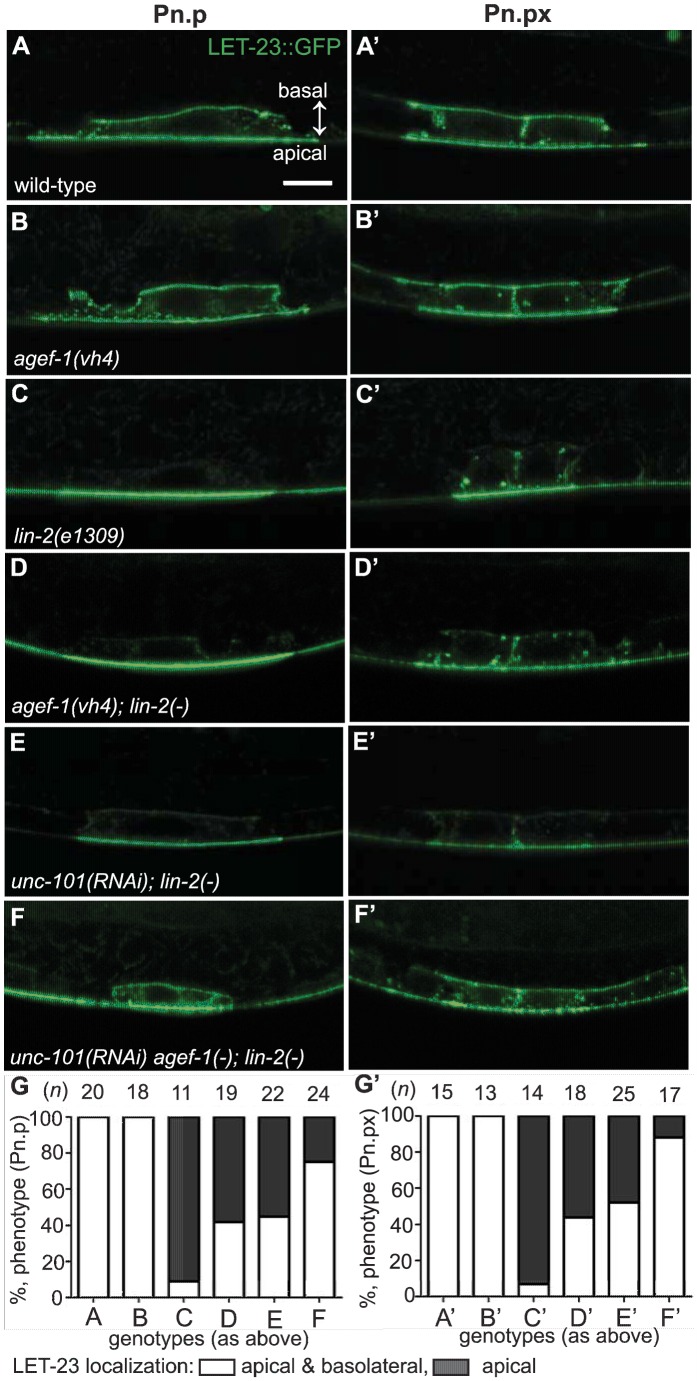
AGEF-1 and UNC-101 AP-1μ antagonize basolateral localization of LET-23 EGFR. (A–F′) Representative confocal images of LET-23::GFP localization in P6.p (A–F) and P6.px (A′–F′) vulval cells of *zhIs035* transgene-carrying animals. (A–B′) The LET-23::GFP is localized to both apical and basolateral membranes in wild-type and *agef-1(vh4)* animals. (C, C′) The basolateral receptor localization is lost in *lin-2(e1309)* mutants. (D, D′) *agef-1(vh4); lin-2(e1309)* mutants with faint LET-23::GFP expression on the basolateral membrane of P6.p and P6.px. (E, E′) *unc-101(RNAi); lin-2(e1309)* animals with faint LET-23::GFP expression on the basolateral membrane of P6.p and P6.px. (F, F′) *unc-101(RNAi)* in *agef-1(vh4); lin-2(e1309)* mutants results in more LET-23::GFP on the basolateral membranes. (G, G′) Percent animals with LET-23::GFP on both the basolateral and apical membranes or apical only localization. Because some animals have no or little basolateral membrane localization, basolateral localization of LET-23::GFP in the P6.p and P6.px cells was determined by measuring the intensity at the basal membrane versus background. If the GFP intensity on the basal membrane was twice that of the background the cell was considered to have basolateral membrane localization. Bar, 5 µm.

The role of AGEF-1 in restricting SID-2::GFP on the apical membrane suggests that AGEF-1, the ARF GTPases and the AP-1 complex might restrict LET-23 EGFR to the apical membrane in the VPCs. To test this hypothesis we made use of two transgenic strains expressing a LET-23 EGFR GFP fusion (*zhIs035* and *zhIs038*) that mimic the localization of endogenous LET-23 EGFR as seen by antibody staining [5]. In wild-type animals, LET-23::GFP localizes to both the apical and basolateral membranes of P6.p and in the *lin-2(e1309)* animals LET-23::GFP localizes strictly to the apical membrane (Figure 5A, C and Figure S4A, C). At the Pn.px stage, some basolateral, or lateral only localization is seen in *lin-2(e1309)* animals. Despite the lack of basolateral localization at the Pn.p stage, we find that the LET-23::GFP transgenes fully rescue the *lin-2(e1309)* Vul phenotype (Table 1, lines 8 and 9), suggesting that the levels of LET-23 EGFR at the basolateral membrane required for VPC induction are below the level of detection. Similarly, the *gaIs27* LET-23::GFP transgene, that is only detectable by immunostaining with anti-GFP antibody, suppressed the *lin-2(e1309)* egg-laying defective phenotype [11].

To determine if AGEF-1 regulates LET-23 EGFR localization we compared the ratio of basolateral versus apical localization of LET-23::GFP in the P6.p cell of wild-type and *agef-1(vh4)* animals (Table 3). In wild-type, the average basal/apical intensity of LET-23::GFP in P6.p was 0.49 for *zhIs035* and 0.65 for *zhIs038*. In *agef-1(vh4)* animals, the average basal/apical intensity of LET-23::GFP in the P6.p cell is 0.79 for *zhIs035* and 0.93 for *zhIs038* reflecting a decrease in apical intensity and an increase in basolateral intensity. We also found LET-23::GFP is present on the basolateral membrane of the intestinal cells in *agef-1(vh4)* animals whereas we did not see this in wild-type by confocal microscopy (Figure S3E–H). Therefore, AGEF-1 represses basolateral localization of LET-23::GFP in the VPCs and intestinal cells.

**Table 3:** AGEF-1 antagonizes basal membrane localization of LET-23 EGFR in P6.p.

Genotype	Avg. Basal Intensity	Avg. Apical Intensity	Avg. Basal/Apical Intensity	*n*
*zhIs035*	1385	2826	0.49	20
*agef-1(vh4) zhIs035*	1548	1959	0.79	18
*zhIs038*	1199	1844	0.65	30
*agef-1(vh4); zhIs038*	1277	1373	0.93	38

We next tested if *agef-1(vh4)* could restore the basolateral localization of LET-23::GFP in *lin-2(1309)* animals. We found that ∼40% of *agef-1(vh4); lin-2(e1309)* animals with *zhIs035* have weak basolateral membrane localization of LET-23::GFP in P6.p compared to 9% in *lin-2(e1309)* animals (Figure 5C–D, G). Similarly, at the P6.px stage, we see an increase in the number of animals with basolateral localization of LET-23::GFP in *agef-1(vh4); lin-2(e1309)* as compared to *lin-2(e1309)* single mutants (Figure 5C′–D′, G′). No basolateral LET-23::GFP was seen with *agef-1(vh4); lin-2(e1309)* animals with the lower expressing *zhIs038* (Figure S4C′–D′, F′). Since *agef-1(vh4)* is a weak hypomorphic mutation, we tested if knocking down the AP-1 complex via *unc-101(RNAi)* can further restore basolateral localization of LET-23 EGFR in *agef-1(vh4)*; *lin-2(e1309)* mutants. We found that *unc-101(RNAi) agef-1(vh4); lin-2(e1309)* animals with either *zhIs035* or *zhIs038* had an increase in basolateral membrane localization of LET-23::GFP in both the intensity and the number of animals (Figure 5F–G′ and Figure S4E, F). *unc-101(RNAi); lin-2(e1309)* animals only showed mild restoration of LET-23::GFP using the *zhIs035* transgene (Figure 5E, E′, G and G′). The restoration of LET-23 EGFR on the basolateral membrane in *agef-1(vh4); lin-2(e1309)*, *unc-101(RNAi); lin-2(e1309)* and *unc-101(RNAi) agef-1(vh4); lin-2(e1309)* animals suggests that AGEF-1 and UNC-101 AP-1μ negatively regulate LET-23 EGFR signaling by limiting basolateral membrane localization.

## Discussion

Regulators of LET-23 EGFR trafficking are likely required for viability, as is the case for the RAB-7 GTPase [11]. In a screen for essential negative regulators of LET-23 EGFR-mediated vulva induction we identified a hypomorphic allele in the *agef-1* gene. AGEF-1 is the *C. elegans* homolog of the yeast Sec7p and human BIG1 and BIG2 Arf GEFs, which function with class I Arf GTPases and the AP-1 complex to regulate cargo sorting and trafficking from the TGN [42]. We demonstrate that AGEF-1 regulates protein secretion, polarized protein localization, and late endosome/lysosome morphology. We show that AGEF-1 antagonizes signaling in the VPCs, upstream or in parallel to LET-23 EGFR, and that the class I/II Arf GTPases, ARF-1.2 and ARF-3, also negatively regulate signaling. Our genetic and phenotypic data are consistent with AGEF-1, the ARF-1.2 and ARF-3 GTPases, and the AP-1 complex together preventing ectopic vulva induction. The AGEF-1/Arf GTPase/AP-1 ensemble antagonizes the basolateral membrane localization of LET-23 EGFR in the VPCs; and hence, LET-23 EGFR-mediated vulva induction.

The clonal screen for suppressors of the *lin-2(e1309)* Vul phenotype was initially aimed at identifying maternal effect lethal mutants, like *rab-7(ok511)*. Instead, we identified two strong suppressors of *lin-2(e1309)* with partial embryonic lethal phenotypes, *agef-1(vh4)* and *vh22* (J. Meng, O.S. and C.E.R., unpublished data); which we currently believe function independently of each other and *rab-7*. The *agef-1* deletion alleles are zygotic lethal and RNAi in the VPCs with *agef-1, arf-1.2* and *rab-7* has proven less effective than their corresponding genetic mutations [11; this study]. Therefore, the *agef-1(vh4)* mutation, being a recessive partial loss-of-function allele, provides a unique tool to study the function of *agef-1*, particularly in tissues refractory to RNAi such as the VPCs and neurons.

The *agef-1(vh4)* lesion changes a conserved negatively charged Glutamic Acid in the HDS2 domain to a positively charged Lysine. Collectively, the HDS2, HDS3, and HDS4 domains of yeast Sec7p have been shown to have an autoinhibitory function [43]. However, the specific function of the HDS2 domain is not known. Given the recessive nature of the *agef-1(vh4)* allele, it suggests that the HDS2 domain has a positive role in promoting AGEF-1 function.

Consistent with yeast Sec7p and human BIG1/BIG2 functioning in the secretory pathway, we found that *agef-1(vh4)* animals had defects in secretion of ssGFP from body wall muscles and Yolk::GFP from the intestine. Similar yolk secretion defects were recently reported for *agef-1(RNAi)* [31]. We found that *agef-1(vh4)* coelomocytes accumulated enlarged LMP-1::GFP positive late endosomes/lysosomes. Independently, Tang *et al.* [27] found that *agef-1(RNAi)* also caused enlargement of LMP-1::GFP vesicles and proposed a role for AGEF-1 in late endosome to lysosome trafficking, however, they did not find a defect in lysosome acidification or protein degradation. We do not know the reason for the enlarged late endosomes/lysosomes, but it could reflect a defect in retrograde transport from late endosomes to the Golgi as has been shown for knockdown of BIG1 and BIG2 or the AP-1 complex in mammalian cells [23]. Consistent with this idea, we found that knockdown of the AP-1 complex also induced enlarged LMP-1::GFP vesicles. Tang *et al.* [27] also reported that ssGFP accumulated in the pseudocoelom suggesting an uptake defect in the coelomocytes. We found that *agef-1(vh4)* mutants accumulated ssGFP in the body wall muscles rather than the pseudocoelom; thus the reduced ssGFP in coelomocytes could be explained by reduced secretion from the body wall muscles. However, we cannot rule out an uptake defect in the coelomocytes as well. Perhaps these discrepancies reflect a difference in reducing the levels of *agef-1* by RNAi versus the *vh4* missense mutation.

Our genetic analysis with *agef-1(vh4)* indicate that AGEF-1 is a potent negative regulator of LET-23 EGFR-mediated vulva induction. Similar to other negative regulators, *agef-1* enhanced the Muv phenotype of the gain-of-function Ras mutant, *let-60(n1046)*, and was a potent suppressor the Vul phenotypes of *lin-2(e1309)* and *let-23(sy1)* mutations, restoring vulva induction and even inducing a Muv phenotype. However, *agef-1(vh4)* failed to suppress a strong *let-23(sy97)* allele similar to *sli-1* and *unc-101* mutations and consistent with a role for AGEF-1 upstream or in parallel to LET-23 EGFR. In accordance with AGEF-1 being an Arf GEF, we found that ARF-1.2 and ARF-3, Class I/II Arf GTPases, also negatively regulate LET-23 EGFR signaling. The *arf-1.2(ok796)* deletion allele was a less potent suppressor of the *lin-2(e1309)* Vul phenotype as compared to the *agef-1(vh4)* mutant. However, *arf-3(RNAi)* in *arf-1.2(ok796); lin-2(e1309)* doubles showed suppression comparable to that in *agef-1(vh4); lin-2(e1309)* mutants. Therefore, ARF-1.2 and ARF-3 appear to function in a partly redundant manner during vulva development. Furthermore, expression of an ARF-1.2::GFP fusion in the VPCs rescued the suppressed Vul phenotype of both *arf-1.2(ok796); lin-2(e1309)* and *agef-1(vh4); lin-2(e1309)* animals indicating that ARF-1.2 antagonizes signaling in the VPCs likely downstream of AGEF-1.

In mammalian cells, the BIG1/BIG2 proteins and Arf1 recruit the AP-1 adaptor protein complex to the TGN and endosomes [15], [19], [20]. Both of the *C. elegans* AP-1μ subunits, *unc-101* and *apm-1*, negatively regulate LET-23 EGFR mediated vulva development [12], [13]. In fact, *apm-1(RNAi) unc-101(sy108)* animals had a Muv phenotype, indicating that UNC-101 and APM-1 are functionally redundant during vulva induction, thus revealing a role for the AP-1 complex in inhibiting ectopic vulva induction [13]. Our findings that various double-mutant combinations between *agef-1(vh4)*, *arf-1.2(ok796)* and *unc-101(sy108) AP-1μ* result in a synergistic Muv phenotype are consistent with AGEF-1, the Arfs and AP-1 functioning together to inhibit ectopic vulva induction. However, we cannot conclude whether they function in parallel pathways or in a common pathway due to the fact that *agef-1(vh4)* is not a null allele and the *unc-101(sy108)* and *arf-1.2(ok796)* mutations, while severe loss-of-function or null alleles, function in a partly redundant manner with *apm-1* and *arf-3*, respectively. We favor a model whereby AGEF-1, the Arfs, and AP-1 function in a common pathway since this is most consistent with data from yeast and mammals, and that loss of AGEF-1 and components of the AP-1 complex have similar phenotypes in coelomocytes and the intestine. While synergistic genetic interactions are typically more indicative of genes in parallel pathways, we interpret that no single mutation in the AGEF-1/Arf/AP-1 pathway is sufficient to increase LET-23 EGFR signaling above a threshold necessary for ectopic induction. It is only when the activity of the AGEF-1/Arf/AP-1 pathway is further compromised by two mutations that LET-23 EGFR signaling increases above a threshold to induce a synergistic Muv phenotype. It is important to note that the AGEF-1/Arf/AP-1 pathway is essential, and only animals that survive to the fourth larval stage can be scored for vulva induction phenotypes. Thus, LET-23 EGFR signaling and localization phenotypes would likely be more severe if we were able to assess true null mutations in the VPCs only.

**Figure 6:**
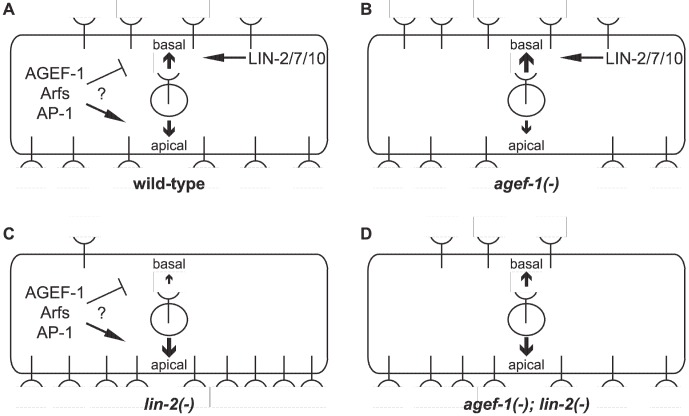
Model of LET-23 EGFR regulation by AGEF-1/Arf/AP-1 and LIN-2/7/10. (A) LET-23 EGFR is localized to both basal and apical membranes in the VPCs of wild-type animals. The LIN-2/7/10 complex promotes the basal localization while the AGEF-1/Arf/AP-1 ensemble either inhibits basal or promotes apical localization. (B) In an *agef-1(-)* background, there is more LET-23 EGFR on the basal and less apical. (C) In a *lin-2(-)* background, most all of the LET-23 EGFR is apical with presumably residue LET-23 EGFR at the basal membrane, but not enough to induce vulva cell fates. (D) In an *agef-1(-); lin-2(-)* background, the loss of AGEF-1 partially restores LET-23 EGFR to the basal membrane sufficient to induce vulva cell fates.

In polarized epithelial cells, the AP-1 complex mediates sorting and polarized distribution of transmembrane proteins, including EGFR, and thus the AGEF-1/Arf GTPase/AP-1 ensemble could regulate signaling via LET-23 EGFR localization. In the P6.p cell, we showed that the localization of LET-23 EGFR is altered in *agef-1(vh4)* animals using two transgenic lines (*zhIs035* and *zhIs038*) expressing LET-23::GFP [5]. In wild-type animals, LET-23::GFP is present on both the apical and basolateral domains, however the average levels of LET-23:GFP on the apical membrane are double (*zhIs035*) or close to double (*zhIs038*) that on the basolateral membrane (Figure 6A). In *agef-1(vh4)* animals there was a redistribution of LET-23::GFP from apical to basolateral membrane bringing the average intensities closer to equal, suggesting that AGEF-1 either promotes apical localization or antagonizes basolateral localization of the receptor (Figure 6A, B). In the *lin-2(e1309)* background, LET-23::GFP is apical only (Figure 6C). In the more highly expressed line, *zhIs035*, we see some lateral only or faint basolateral in the P6.p descendants, P6.pa and P6.pp of *lin-2(e1309)* larvae. In the *zhIs035* line, *agef-1(vh4)* partially restores LET-23::GFP on the basolateral membrane in *lin-2(e1309)* larvae. RNAi of *unc-101* also partially restores basolateral localization and enhances the effect of *agef-1(vh4)* such that we see increased levels of LET-23::GFP, with both lines, in *lin-2(e1309)* larvae (Figure 6D). Therefore, AGEF-1 and UNC-101 AP-1μ cooperate to antagonize LET-23 EGFR basolateral localization and thus provide a mechanism by which these genes/proteins antagonize LET-23 EGFR signaling. Despite the lack of basolateral localization of LET-23::GFP in *lin-2* mutant animals, the two LET-23::GFP transgenes used in this study rescued the *lin-2(e1309)* Vul phenotype, suggesting that the levels of receptor required for VPC induction are below detection. Therefore, the modest amount of LET-23::GFP restored to the basolateral membrane in *agef-1(vh4); lin-2(e1309)* or *unc-101(RNAi); lin-2(e1309)* could be more than sufficient to explain the strong restoration of VPC induction in these double mutants.

Our findings that an AGEF-1/Arf GTPase/AP-1 ensemble antagonizes the basolateral localization of LET-23 EGFR is contradictory to the established role of the mammalian AP-1A and AP-1B complexes in sorting transmembrane proteins to the basolateral membrane through the specific binding of basolateral sorting motifs in the cytoplasmic tail [18]. In fact, the AP-1B complex promotes the basolateral localization of EGFR in MDCK cells [17]. LET-23 EGFR does have several putative AP-1 sorting motifs, and thus could be a direct target for AP-1 regulation, but this would imply that AP-1 is impeding basolateral localization. A precedent for AP-1 having an antagonistic role in protein sorting or secretion has been found with the yeast Chs3p and Fus1p proteins, which rely on the exomer for secretion [44], [45]. In the absence of exomer, Chs3p and Fus1p are retained internally in an AP-1 dependent manner [45], [46], [47]. An analogous situation whereby the LIN-2/7/10 complex sorts/maintains LET-23 EGFR localization on the basolateral membrane and the AGEF-1/Arf/AP-1 pathway plays an antagonistic role could exist.

Recent studies in *C. elegans* and mice have shown that both basolateral and apical membrane cargos are mislocalized in the absence of the AP-1 complex [39], [40], [48], suggesting that the AP-1 complex is required to maintain the polarity of the epithelial cells [reviewed in 18]. Similarly, we find that *agef-1(vh4)* mutants mislocalized the SID-2 protein to the basolateral membranes, which is strictly apical in wild-type animals. Therefore, AGEF-1 might function with AP-1 to maintain polarity in the intestinal epithelia and by extension the AGEF-1/Arf GTPase/AP-1 ensemble could indirectly regulate LET-23 EGFR localization via maintenance of VPC polarity.

In summary, an AGEF-1/Arf GTPase/AP-1 ensemble functions opposite the LIN-2/7/10 complex to regulate apical versus basolateral localization of LET-23 EGFR in the VPCs, thus explaining how it negatively regulates LET-23 EGFR-mediated vulva induction. We don't yet know whether the AGEF-1/Arf GTPase/AP-1 ensemble directly regulates LET-23 EGFR sorting and localization or whether it is indirect via maintenance of VPC polarity. Further studies will be required to sort out the mechanisms by which the AGEF-1/ARF GTPase/AP-1 ensemble regulates LET-23 EGFR localization.

## Materials and Methods

### Strains and alleles

General methods for the handling and culturing of *C. elegans* were as previously described [49]. *C. elegans* Bristol strain N2 is the wild-type parent for all the strains used in this study; *E. coli* stain HB101 was used as a food source. The Hawaiian strain CB4856 was used for SNP mapping. All experiments were performed at 20°C. Information on the genes and alleles used in this work can be found on WormBase (www.wormbase.org) and are available through Caenorhabditis Genetics Center (www.cbs.umn.edu/cgc) unless otherwise noted in the strain list (Table S1).

### *lin-2(e1309)* suppressor screen

*lin-2(e1309)* L4 hermaphrodites were mutagenized as previously described [49]. F1 progeny (*m/+; lin-2*) were transferred to individual plates. Due to the strong Vul phenotype of *lin-2(e1309)* animals, the self-progeny hatch internally [6]. F2 progeny were screened at the adult stage in order to identify plates that had a large number of eggs and egg-layers, additional preference was given to plates that had Muv, embryonic lethality, or dumpy phenotypes, similar to the *rab-7(ok511)* mutant. Progeny of a total of 2430 F1 animals (4860 haploid genomes) were screened and two *lin-2 (e1309)* suppressor mutants that are dumpy and partly embryonic lethal were identified, *vh4* and *vh22*.

### Genetic mapping and cloning of *agef-1(vh4)*

Single nucleotide polymorphism (SNP) mapping was used to place *vh4* to the right arm of chromosome I [25]. Chromosome mapping showed linkage of *vh4* to SNPs at 13 (F58D5), 14 (T06G6) and 26 (Y105E8B) map units (m.u.). Interval mapping using two sets of recombinants, 141 animals in total, was conducted using the following SNPs: *pkP1133* at 17.4 m.u (A/T Bristol/CB4856, RFLP DraI); *pkP1134* at 18.95 m.u. (T/C Bristol/CB4856, RFLP AflIII); *haw14061* at 19.51 m.u. (T/C Bristol/CB4856, sequencing); *haw14137* at 20.65 m.u. (T/A Bristol/CB4856, sequencing); *haw14164* at 21.04 m.u. (C/T Bristol/CB4856, sequencing); *CE1-248* at 21.97 m.u. (T/A Bristol/CB4856, sequencing); *CE1-220* at 23.16 m.u. (A/G Bristol/CB4856, sequencing); *pkP1071* at 23.4 m.u. (C/T Bristol/CB4856, RFLP EcoRI). In the course of interval mapping the following predicted sequencing SNPs were confirmed: *haw14061* and *haw14137* as T/C and T/A Bristol/CB4856, respectively. Genomic DNA from *vh4* and *vh22* was isolated and submitted to Genome Quebec for Illumina sequencing. Within the defined map region, the *agef-1* gene was the only gene carrying a non-synonymous mutation unique to the *vh4* strain.

### RNA interference

RNAi feeding was performed essentially as previously described [50] using the *unc-101* (I-6G20), *agef-1* (I-6L22), *arf-1.2* (III-3A13), and *arf-3* (IV-4E13) clones from Ahringer RNAi library (Geneservice, Cambridge, United Kingdom). Clones were verified by DNA sequencing. To avoid embryonic and larval lethal phenotypes, synchronized L1 larvae were placed on RNAi plates and scored for vulva induction when the animals reached L4 stage 36–48 hours later.

### Microscopy and phenotype analysis

General methods for live animal imaging using Nomarski differential interference contrast (DIC) microscopy were as previously described [51]. Animals were analyzed on an Axio Zeiss A1 Imager compound microscope (Zeiss, Oberkochen, Germany) and images were captured using an Axio Cam MRm camera and AxioVision software (Zeiss, Oberkochen, Germany). Muv and Vul phenotypes were scored by counting the numbers of vulval and non-vulval descendants of P3.p–P8.p in L4 stage larvae as described previously [11]. Fisher's exact test (www.graphpad.com/quickcalcs) was used for statistical analysis of the vulval phenotypes. Comparison of GFP intensities wild-type and *agef-1(vh4)* was performed using identical exposure times for conditions being compared. Fiji image processing tool was used to measure intensities in raw images; any adjustments to contrast/brightness were for presentation purposes and were performed after analysis [52]. Tissue/organ of interest was outlined using free hand selection tool followed by measurement of the average pixel intensity. Images selected for figures are representative of the mean value for average pixel intensity for the group. Statistical analysis and graphing was done using Prism 5 (GraphPad Software, Inc., La Jolla, CA).

Confocal analysis was performed using a Zeiss LSM-510 Meta laser scanning microscope with 63× oil immersion lens (Zeiss, Oberkochen, Germany) in a single-track mode using a 488 nm excitation for GFP. Images were captured using ZEN 2009 Image software (Zeiss, Oberkochen, Germany). Animals at the L4 larval stage were selected for visualization of endocytic/secretory compartments in the coelomocytes. Images selected for figures are representative of the mean value for the largest vesicle diameter for the group. Statistical analysis and graphing was done using Prism 5 (GraphPad Software, Inc., La Jolla, CA). Confocal analysis of *zhIs038* transgene-carrying animals was performed at early L3 larval stage using the Zeiss LSM-510 Meta laser scanning microscope. Confocal analysis of *zhIs035* was performed using the Zeiss Axio Observer Z1 LSM-780 laser scanning microscope with 63× oil immersion lens (Zeiss, Oberkochen, Germany) in a single-track mode using an Argon multiline laser with 488 nm excitation for GFP. Images were captured using ZEN 2010 Image software (Zeiss, Oberkochen, Germany). The apical and basal LET-23::GFP intensities were measured using Fiji by drawing a line through the center of the nucleus in the DIC channel and transferring the selection into the GFP channel to prevent bias.

### Plasmid and transgenic construction

*arf-1.2* was amplified by PCR from wild-type cDNA using the primers 5′-CATAAGAATAGTCGACATGGGAAACGTGTTCGGCAGC-3′ (forward) and 5′-GATTCTGATTACCGGTTCAGATCTATTCTTGAGCT-3′ (reverse) containing SalI and AgeI cut sites, respectively. The PCR product was cloned into pEGFP-N1 plasmid using SalI 639 and AgeI 666 sites. *arf-1.2::GFP* was digested using SalI and NotI and subcloned into the p255 *lin-31* promoter plasmid. Transgenic animals were generated by DNA microinjection [53] of the *Plin-31::ARF-1.2::GFP* plasmid and a marker plasmid *Pttx-3::GFP* at a concentration of 50 ng/µl of each into N2 animals using maxiprep quality DNA. Two of three lines were used for this study, *vhEx7* and *vhEx8*. Rescue of *agef-1(vh4)* and *arf-1.2(ok796)* mediated suppression of the *lin-2(e1309)* Vul phenotype was scored in animals expressing *ARF-1.2::GFP* in the VPCs.

### Phylogenetic analysis

Analysis of the Arf GTPases was performed using MAFFT version 7 multiple alignment program for amino acid or nucleotide sequences online (http://mafft.cbrc.jp) [54]. Input sequences were human NP_001649.1 (Arf1), NP_001650.1 (Arf3), NP_001651.1 (Arf4), NP_001653.1 (Arf5), AAV38671.1 (Arf6), NP_001168.1 (Arl1) and *C. elegans* NP_501242.1 (ARF-1.1), NP_498235.1 (ARF-1.2), NP_501336.1 (ARF-3), NP_503011.1 (ARF-6), NP_495816.1 (ARL-1). Phylogenetic tree was constructed and visualized using Archaeopteryx [55], [56].

## Supporting Information

Figure S1 *agef-1(vh4)* animals are defective in Yolk secretion from the intestine and CAV-1 body formation in oocytes. (A–D) DIC and corresponding epifluorescent images (55 ms exposure time) of the oocytes of wild-type and *agef-1(vh4)* animals expressing YP170::GFP. (E–H) Representative DIC and corresponding epifluorescent images (50 ms exposure time) of the intestine of wild-type and *agef-1(vh4)* animals expressing YP170::GFP. (I) Quantification of the mean YP170::GFP pixel intensity in the intestine. Statistical analysis was performed as described in Figure 4. (J–M) Epifluorescent images (80 ms exposure time) of wild-type and *agef-1(vh4)* oocytes expressing CAV-1::GFP. The areas outlined with white squares in (J) and (L) are enlarged in (K) and (M), respectively. CAV-1::GFP forms ring-like structures, CAV-1 bodies, in wild-type animals (J, K), which are largely absent from *agef-1(vh4)* mutant oocytes (L, M). All bars, 10 µm.
(TIF)

Figure S2 *agef-1(vh4)* mutants have enlarged endosomal compartments. Representative confocal images of wild-type (A, D) and *agef-1(vh4)* (B, E) coelomocytes expressing either RME-8::GFP endosomal or MANS::GFP Golgi markers. (C, F) Quantification of the diameter of the largest GFP-positives structures demonstrate an increase in the size of endosomes outlined by RME-8::GFP. Statistical analysis is the same as in Figure 3. Bar, 5 µm.
(EPS)

Figure S3 AGEF-1 antagonizes basolateral localization of SID-2::GFP and LET-23::GFP in the intestine. (A–D) Representative DIC and epifluorescent images of the intestine of wild-type and *agef-1(vh4)* animals expressing SID-2::GFP. The arrows mark the intestinal lumen corresponding to the apical membrane of the intestinal cells. Note that in wild-type animals SID-2::GFP expression is restricted to the apical membrane, whereas in *agef-1(vh4)* mutants SID-2::GFP is localized to both apical and basolateral membranes. (E, G) Confocal images of the intestine of wild-type and *agef-1(vh4)* animals carrying the *zhIs035* transgene. LET-23::GFP is present on the basolateral membrane of intestinal cells in *agef-1(vh4)* mutants, but is not detected in wild-type animals. (F, H) Graphs indicate the fluorescent intensity along a line drawn across the intestine in wild-type and *agef-1(vh4)* animals. The two distinct intensity peaks observed in (H) mark LET-23::GFP on the basolateral membrane in (G). All bars, 20 µm.
(TIF)

Figure S4 AGEF-1 and UNC-101 AP-1μ antagonize basolateral localization of LET-23 EGFR. (A–E′) Confocal images of *zhIs038* LET-23::GFP in the P6.p (A–E) and P6.px (A′–E′) cells. (A–B′) Both wild-type and *agef-1(vh4)* animals express LET-23::GFP on both the basolateral and apical membranes. (C, C′) Basolateral localization is lost in *lin-2(e1309)* mutants. (D, D′) *agef-1(vh4); lin-2(e1309)* double mutants do not have detectable basolateral LET-23::GFP. (E, E′) *unc-101(RNAi)* in *agef-1(vh4); lin-2(e1309)* background results in faint basolateral LET-23::GFP accumulation. (F, F′) Quantification of LET-23::GFP localization in P6.p and P6.px cells. Bar, 5 µm.
(EPS)

Table S1 Strain list. Names and genotypes of strains use in this study. Unless otherwise noted strains were obtained from the Caenorhabditis Genetics Center (http://www.cbs.umn.edu/research/resources/cgc).
(PDF)
