# Supplementary figures and images for: An AGEF-1/Arf GTPase/AP-1 Ensemble Antagonizes LET-23 EGFR Basolateral Localization and Signaling during C. elegans Vulva Induction
Source: PLoS Genet. 2014 Oct 16;10(10):e1004728. doi: 10.1371/journal.pgen.1004728 (PMC4199573; doi:10.1371/journal.pgen.1004728)

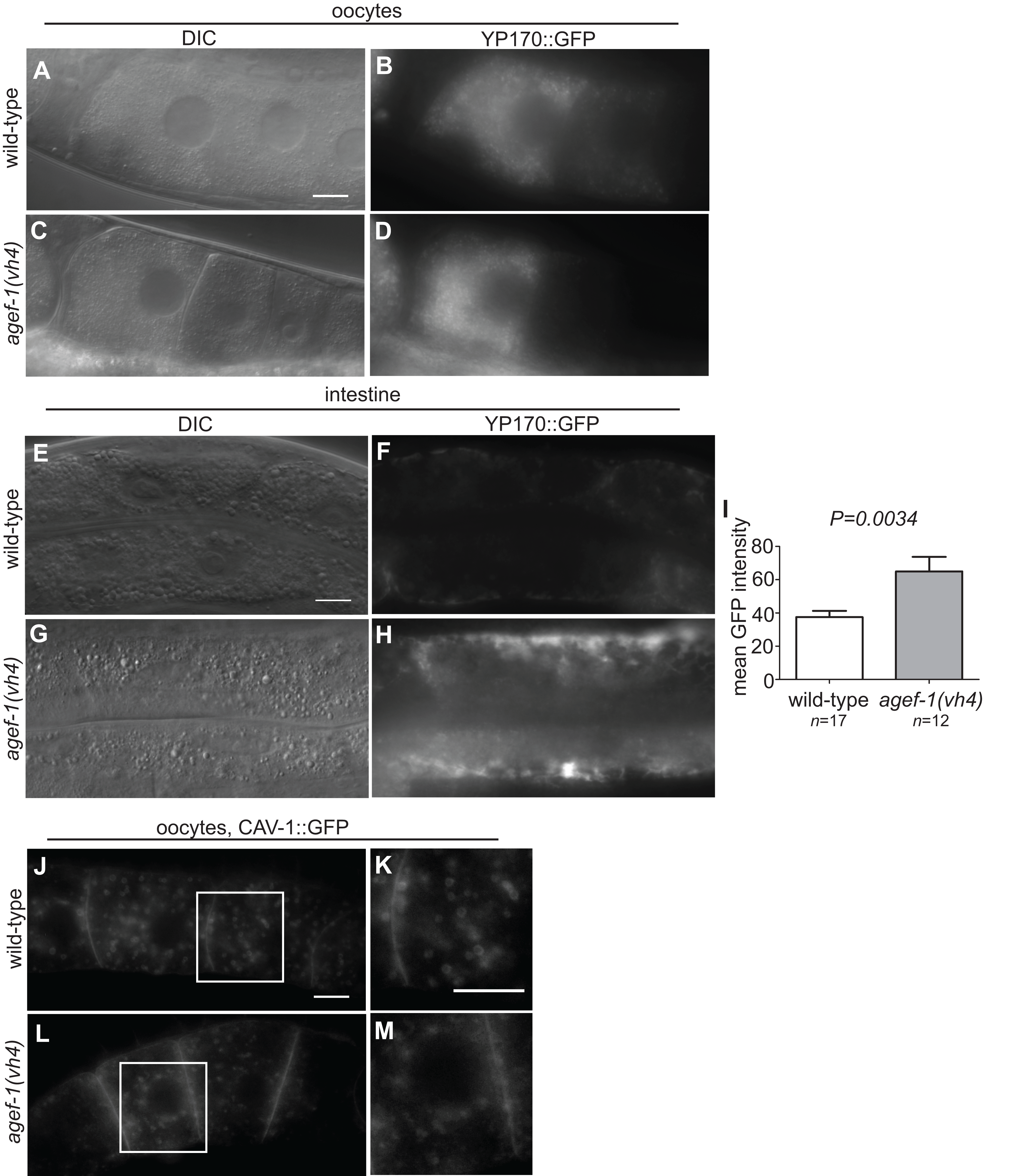

Supplement: Figure S1 — agef-1(vh4) animals are defective in Yolk secretion from the intestine and CAV-1 body formation in oocytes. (A–D) DIC and corresponding epifluorescent images (55 ms exposure time) of the oocytes of wild-type and agef-1(vh4) animals expressing YP170::GFP. (E–H) Representative DIC and corresponding epifluorescent images (50 ms exposure time) of the intestine of wild-type and agef-1(vh4) animals expressing YP170::GFP. (I) Quantification of the mean YP170::GFP pixel intensity in the intestine. Statistical analysis was performed as described in Figure 4. (J–M) Epifluorescent images (80 ms exposure time) of wild-type and agef-1(vh4) oocytes expressing CAV-1::GFP. The areas outlined with white squares in (J) and (L) are enlarged in (K) and (M), respectively. CAV-1::GFP forms ring-like structures, CAV-1 bodies, in wild-type animals (J, K), which are largely absent from agef-1(vh4) mutant oocytes (L, M). All bars, 10 µm. (TIF) [file pgen.1004728.s001.tif]

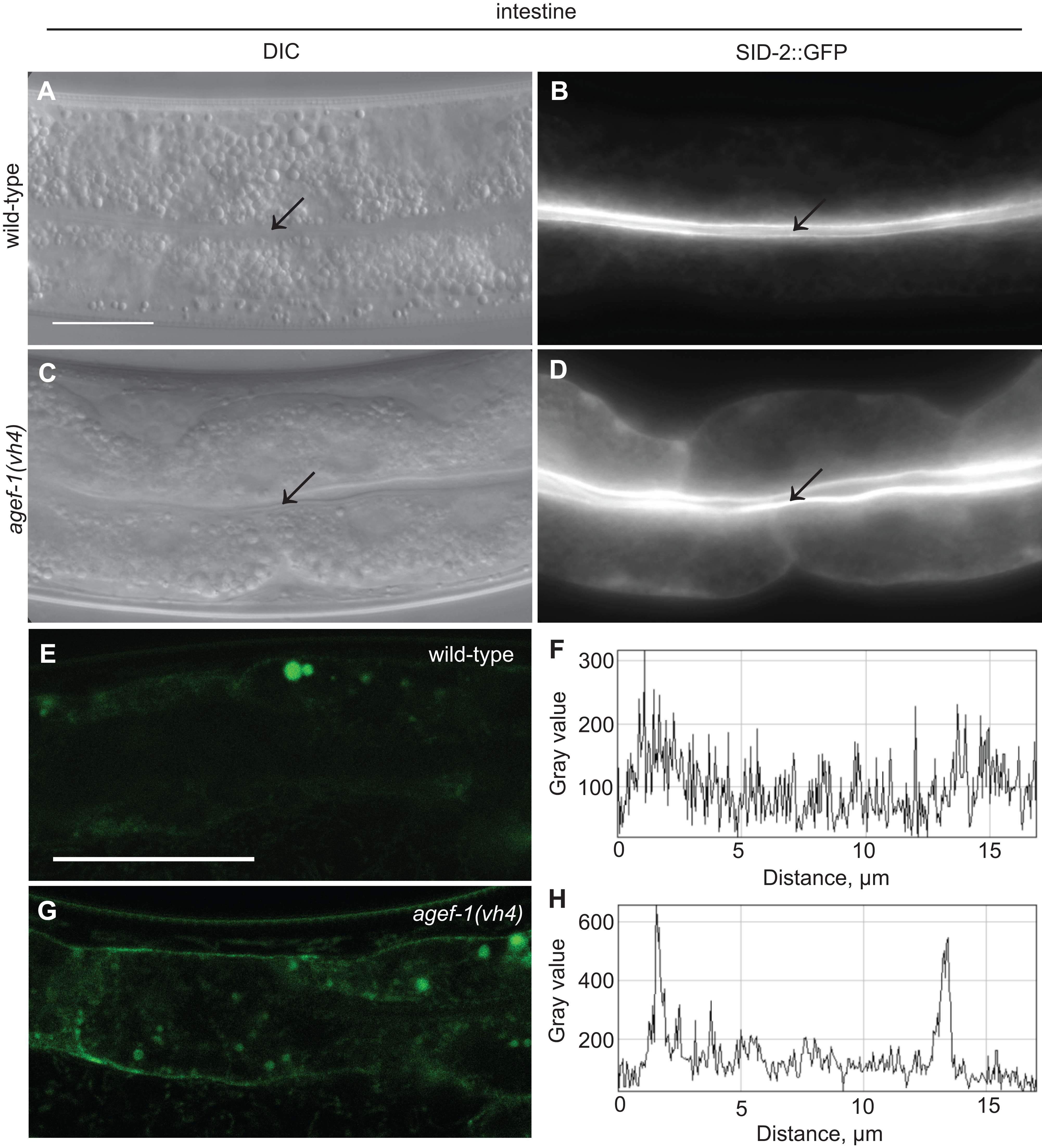

Supplement: Figure S3 — AGEF-1 antagonizes basolateral localization of SID-2::GFP and LET-23::GFP in the intestine. (A–D) Representative DIC and epifluorescent images of the intestine of wild-type and agef-1(vh4) animals expressing SID-2::GFP. The arrows mark the intestinal lumen corresponding to the apical membrane of the intestinal cells. Note that in wild-type animals SID-2::GFP expression is restricted to the apical membrane, whereas in agef-1(vh4) mutants SID-2::GFP is localized to both apical and basolateral membranes. (E, G) Confocal images of the intestine of wild-type and agef-1(vh4) animals carrying the zhIs035 transgene. LET-23::GFP is present on the basolateral membrane of intestinal cells in agef-1(vh4) mutants, but is not detected in wild-type animals. (F, H) Graphs indicate the fluorescent intensity along a line drawn across the intestine in wild-type and agef-1(vh4) animals. The two distinct intensity peaks observed in (H) mark LET-23::GFP on the basolateral membrane in (G). All bars, 20 µm. (TIF) [file pgen.1004728.s003.tif]
