# Supplementary material for: An AGEF-1/Arf GTPase/AP-1 Ensemble Antagonizes LET-23 EGFR Basolateral Localization and Signaling during C. elegans Vulva Induction
Source: PLoS Genet. 2014 Oct 16;10(10):e1004728. doi: 10.1371/journal.pgen.1004728 (PMC4199573; doi:10.1371/journal.pgen.1004728)
Supplement: Table S1 — Strain list. Names and genotypes of strains use in this study. Unless otherwise noted strains were obtained from the Caenorhabditis Genetics Center (http://www.cbs.umn.edu/research/resources/cgc). (PDF) [file pgen.1004728.s005.pdf]

Table S1. Strain list

CB1309 *lin-2(e1309)* X
CB1417 *lin-3(e1417)* IV
CB2769 *eDf3/eDf24* I
CB4856 (Hawaiian mapping strain)
DH1033 *sqt-1(sc103)* II*;bIs1[Pvit-2::VIT-2::GFP; rol-6(su1006)]* X
DH1336 *bIs34[Prme-8::GFP::RME-8; rol-6]*
FX01447 *arf-6 (tm1447)* IV (Shohei Mitani, Tokyo Women's Medical University)
FX01693 *agef-1(tm1693)/+* I (Shohei Mitani, Tokyo Women's Medical University)
FX03767 *vps-28(tm3767)/+* I (Shohei Mitani, Tokyo Women's Medical University)
FX12006 *arf-3(tm1877)/nT1* (Shohei Mitani, Tokyo Women's Medical University)
GS1912 *arIs37[pmyo-3::ssGFP; dpy-20(+)]* I*; dpy-20(e1282)* IV
HC722 *qtIs5[SID-2::C-GFP]; sid-2(gk505)* III (Craig Hunter, Harvard University)
MT2124 *let-60(n1046)* IV
N2 (Bristol wild-type parent strain)
NP822 *unc-119(ed3)* III*; cdIs54[pcc-1::MANS::GFP; unc-119(+)]*
NP941 *unc-119(ed3)* III*; cdIs85 [pcc-1::2xFYVE::GFP + unc-119(+) + Pmyo-2::GFP]*
PS80 *let-23(sy1) unc-4(e120)* II
PS295 *let-23(sy97) unc-4(e120)/mnC1[dpy-10(e128) unc-152(e444)]* II
PS529 *unc-101(sy108)* I
PS2728 *sli-1(sy143)* X
QR180 *agef-1(vh4)* I (this study)
QR201 *agef-1(vh4)* I; *bIs1[Pvit-2::VIT-2::GFP; rol-6(su1006)]* (this study)
QR202 *agef-1(vh4)* I*; pwIs50[Plmp-1::LMP-1::GFP + Cbr-unc-119(+)]* (this study)
QR252 *agef-1(vh4)* I; *pwIs28(Ppie-1::CAV-1::GFP)* (this study)
QR268 *arf-1.2(ok796)* III*; lin-2(e1309)* X (this study)
QR269 *agef-1(vh4)* I; *bIs34[Prme-8::GFP::RME-8; rol-6]* (this study)
QR277 *agef-1(vh4)* I; *lin-3(e1417)* IV (this study)
QR293 *agef-1(vh4)* I*; let-23(sy97) unc-4(e120)/mIn1* II (this study)
QR297 *agef-1(vh4)* I*; let-60(n1046)* IV
QR301 *agef-1(vh4)* I; *qtIs5(SID-2::C-GFP)* (this study)
QR307 *agef-1(vh4)* I*; let-23(sy1) unc-4(e120)* II (this study)
QR324 *agef-1(vh4)* I; *cdIs54[pcc-1::MANS::GFP; unc-119(+); myo2::GFP]* (this study)
QR325 *arf-1.1 and F45E4.7(ok1840)* IV*; lin-2(e1309)* X (this study)
QR326 *agef-1(vh4)* I*; cdIs85 [pcc-1::2xFYVE::GFP + unc-119(+) + Pmyo-2::GFP]* (this study)
QR339 *agef-1(vh4)* I*; sli-1(sy143)* X
QR344 *arIs37[pmyo-3::ssGFP; dpy-20(+)] agef-1(vh4)* I (this study)
QR347 *arf-6(tm1447)* IV*; lin-2(e1309)* X (this study)
QR356 *agef-1(vh4)* I*; arf-1.2(ok796)/hT2* III (this study)
QR357 *unc-101(sy108)* I*; arf-1.2(ok796)* III (this study)
QR382 *arf-1.2(ok796)* III*; lin-2(e1309)* X; *vhEx7[Plin-31::ARF-1.2::GFP + Pttx-3::GFP]* (this study)
QR383 *arf-1.2(ok796)* III*; lin-2(e1309)* X; *vhEx8[Plin-31::ARF-1.2::GFP + Pttx-3::GFP]* (this study)

QR401 *unc-101(sy108)* I; *pwIs50[Plmp-1::LMP-1::GFP + Cbr-unc-119(+)]* (this study)
QR403 *agef-1(vh4)* I*; lin-2(e1309)* X*; vhEx7[Plin-31::ARF-1.2::GFP + Pttx-3::GFP]* (this study)
QR404 *agef-1(vh4)* I*; lin-2(e1309)* X*; vhEx8[Plin-31::ARF-1.2::GFP + Pttx-3::GFP]* (this study)
QR475 *agef-1(vh4)* I*; zhIs038[Plet-23::LET-23::GFP; unc-119(+)]* IV (this study)
QR476 *zhIs038[Plet-23::LET-23::GFP; unc-119(+)]; lin-2(e1309)* X (this study)
QR477 *agef-1(vh4)* I; *zhIs038[Plet-23::LET-23::GFP; unc-119(+)]* IV*; lin-2(e1309)* X (this study)
QR479 *zhIs035[Plet-23::LET-23::GFP; unc-119(+)] agef-1(vh4)* I (this study)
QR480 *zhIs035[Plet-23::LET-23::GFP; unc-119(+)]* I*; lin-2(e1309)* I (this study)
QR512 *agef-1(vh4)* I; *lin-2(e1309)* X (this study)
QR513 *zhIs035[Plet-23::LET-23::GFP; unc-119(+)] agef-1(vh4)* I*; lin-2(e1309)* X (this study)
RB1535 *arf-1.1 and F45E4.7(ok1840)* IV
RT258 *unc-119(ed3)* III*; pwIs50[Plmp-1::LMP-1::GFP + Cbr-unc-119(+)]*
RT688 *unc-119(ed3)* III; *pwIs28[Ppie-1::CAV-1::GFP + unc-119(+)]*
SL536 *dxDf2/spe-9(eb19) unc-101(m1)* I
VC567 *arf-1.2(ok796)* III
VC1286 *agef-1(ok1736)/hIn1[unc-101(sy241)]* I
zhIs035[Plet-23::LET-23::GFP; unc-119(+)] I (Alex Hajnal, University of Zurich)
zhIs038[Plet-23::LET-23::GFP; unc-119(+)] III (Alex Hajnal, University of Zurich)
